# di-Cysteine motifs in the C-terminus of plant HMA4 proteins confer nanomolar affinity for zinc and are essential for HMA4 function *in vivo*

**DOI:** 10.1093/jxb/ery311

**Published:** 2018-08-22

**Authors:** Gilles Lekeux, Clémentine Laurent, Marine Joris, Alice Jadoul, Dan Jiang, Bernard Bosman, Monique Carnol, Patrick Motte, Zhiguang Xiao, Moreno Galleni, Marc Hanikenne

**Affiliations:** 1 InBioS – Center for Protein Engineering (CIP), Biological Macromolecules, University of Liège, Liège, Belgium; 2 InBioS – PhytoSystems, Functional Genomics and Plant Molecular Imaging, University of Liège, Liège, Belgium; 3 InBioS – PhytoSystems, Laboratory of Plant and Microbial Ecology, Department of Biology, Ecology, Evolution, University of Liège, Liège, Belgium; 4 School of Chemistry and Bio21 Molecular Science and Biotechnology Institute, The University of Melbourne, Parkville, Victoria, Australia

**Keywords:** Arabidopsis, HMA4, hyperaccumulation, *in vivo* imaging, metal binding, metal–protein interaction, P-type ATPase, zinc

## Abstract

The P_IB_ ATPase heavy metal ATPase 4 (HMA4) has a central role in the zinc homeostasis network of *Arabidopsis thaliana*. This membrane protein loads metal from the pericycle cells into the xylem in roots, thereby allowing root to shoot metal translocation. Moreover, HMA4 is key for zinc hyperaccumulation as well as zinc and cadmium hypertolerance in the pseudometallophyte *Arabidopsis halleri*. The plant-specific cytosolic C-terminal extension of HMA4 is rich in putative metal-binding residues and has substantially diverged between *A. thaliana* and *A. halleri*. To clarify the function of the domain in both species, protein variants with truncated C-terminal extension, as well as with mutated di-Cys motifs and/or a His-stretch, were functionally characterized. We show that di-Cys motifs, but not the His-stretch, contribute to high affinity zinc binding and function *in planta*. We suggest that the HMA4 C-terminal extension is at least partly responsible for protein targeting to the plasma membrane. Finally, we reveal that the C-terminal extensions of both *A. thaliana* and *A. halleri* HMA4 proteins share similar function, despite marginally different zinc-binding capacity.

## Teaser

Functional analysis of the HMA4 protein C-terminal extension reveals a similar and key function of high affinity zinc binding by di-Cys motifs in both *Arabidopsis thaliana* and *A. halleri*.

## Introduction

Zinc is fundamental for all forms of life, including plants (Broadley *et al.*, 2007; Hänsch and Mendel, 2009). This transition metal is widely used in proteins where it has structural or catalytic roles (Auld, 2001; Andreini *et al.*, 2008). It is estimated that the percentage of the genome encoding Zn^2+^-binding proteins ranges from 4% in bacteria to 10% in eukaryotes (Andreini *et al.*, 2006). However, high concentrations of zinc and other essential transition metals (e.g. copper and iron), as well as some metals known as non-essential (e.g. cadmium, lead, and mercury), are toxic for cells (Goyer, 1997; Nzengue *et al.*, 2011; Lemire *et al.*, 2013; Clemens and Ma, 2016). Plants have therefore developed a metal homeostasis network allowing a tight control of metal availability at both the organism and the cellular level, consisting of uptake, chelation, compartmentalization, and efflux mechanisms (Krämer and Clemens, 2005; Palmer and Guerinot, 2009).

Heavy metal ATPase 4 (HMA4) is an essential component of the zinc homeostasis network in *Arabidopsis thaliana* (Mills *et al.*, 2003; Hussain *et al.*, 2004; Verret *et al.*, 2004; Wong and Cobbett, 2009; Cun *et al.*, 2014). In this species, eight HMA proteins have been identified (Axelsen and Palmgren, 2001; Williams and Mills, 2005; Pedersen *et al.*, 2012; Hanikenne and Baurain, 2014). HMA5–8 are monovalent cation pumps involved in copper homeostasis (Woeste and Kieber, 2000; Shikanai *et al.*, 2003; Abdel-Ghany *et al.*, 2005; Andrés-Colás *et al.*, 2006; Kobayashi *et al.*, 2008; Binder *et al.*, 2010). HMA1 is a broad-specificity divalent cation transporter (Ca^2+^, Cd^2+^, Zn^2+^, Cu^2+^, Co^2+^) located in the chloroplast (Seigneurin-Berny *et al.*, 2006; Moreno *et al.*, 2008; Kim *et al.*, 2009; Boutigny *et al.*, 2014), whereas HMA3 localizes to the vacuolar membrane and is involved in zinc/cadmium sequestration (Gravot *et al.*, 2004; Morel *et al.*, 2009). Finally, HMA2 and HMA4 are both found in the plasma membrane and are expressed in root pericycle and in shoot cells bordering the xylem (Hussain *et al.*, 2004; Verret *et al.*, 2004; Sinclair *et al.*, 2007; Wong *et al.*, 2009; Siemianowski *et al.*, 2013). Together, HMA2 and HMA4 are responsible for zinc and cadmium loading in root xylem, thereby allowing their translocation from root to shoot (Hussain *et al.*, 2004; Verret *et al.*, 2004; Wong and Cobbett, 2009; Cun *et al.*, 2014). They are also essential for zinc loading in seeds (Olsen *et al.*, 2016). An *hma2hma4* double mutant has a stunted growth phenotype, resulting from severe zinc deficiency in shoots (Hussain *et al.*, 2004). In addition, HMA4 plays a key role in zinc and cadmium hyperaccumulation and hypertolerance in the pseudometallophyte *Arabidopsis halleri* (Courbot *et al.*, 2007; Willems *et al.*, 2007; Hanikenne *et al.*, 2008; Verbruggen *et al.*, 2009; Krämer, 2010; Hanikenne and Nouet, 2011). In this species, *HMA4* is overexpressed thanks to *cis*-activation and triplication of the gene, which triggers higher rates of root-to-shoot metal translocation compared with *A. thaliana* (Talke *et al.*, 2006; Hanikenne *et al.*, 2008).

HMA proteins are P_IB_ ATPases whose protein architecture consists of a transmembrane domain (TM domain) and two cytoplasmic catalytic domains, the actuator domain (A domain) and the ATP-binding domain (ATP domain). The latter is divided into a nucleotide-binding domain (N domain) and a phosphorylation domain (P domain). In addition, most of them possess N-, as well as occasionally C-, terminal cytosolic extensions (Williams and Mills, 2005; Argüello *et al.*, 2007; Rosenzweig and Argüello, 2012). P_IB_ ATPases are a subfamily of the larger P-type ATPase family that are membrane proteins coupling ATP hydrolysis to their transport of substrate, following the E1/E2 Post–Albers cycle (Albers *et al.*, 1963; Post and Sen, 1965). During this cycle, the phosphorylation and dephosphorylation of an invariant Asp residue located in the P domain, as well as ion binding in the TM domain, trigger conformational changes allowing ion transport across the membrane (Kühlbrandt, 2004; Palmgren and Nissen, 2011; Rosenzweig and Argüello, 2012; Sitsel *et al.*, 2015). The TM domain of P_IB_ ATPases is involved in the metal specificity of the transporter (Argüello, 2003; Williams and Mills, 2005; Hanikenne and Baurain, 2014; Smith *et al.*, 2014). A recent study combining homology modeling of the *A. thaliana* HMA4 TM region to functional analysis *in vivo* delineated a zinc permeation pathway across the membrane (G. Lekeux *et al*., unpublished results). Similar to HMA2 (Eren *et al.*, 2007; Wong *et al.*, 2009), the CCxxE metal binding motif present in the HMA4 N-terminal extension binds Zn^2+^ with nanomolar affinity and this interaction is essential for the function of the protein *in planta* (Zimmermann *et al.*, 2009; Laurent *et al.*, 2016). The HMA4 N-terminal metal binding domain might achieve its function through interaction with a docking platform positioned at the membrane interface of the TM domain (G. Lekeux *et al*., unpublished results) as suggested for its prokaryotic homolog ZntA of *Shigella sonnei* (Wang *et al.*, 2014*a*).

In contrast to their bacterial homologs, the well-known ZntA and CadA efflux pumps (Nucifora *et al.*, 1989; Rensing *et al.*, 1997), plant zinc/cadmium P_IB_ ATPases exhibit a cytosolic C-terminal extension that is rich in putative metal-binding amino acids such as Cys, His, Asp, and Glu residues (Williams and Mills, 2005; Argüello *et al.*, 2007; Rosenzweig and Argüello, 2012). Interestingly, they all possess di-Cys motifs ranging from two in AtHMA3 to 13 in AtHMA4 (Williams and Mills, 2005). The AtHMA2 C-terminal extension (HMA2c), with six di-Cys motifs (Supplementary Fig. S1 at *JXB* online), was shown to bind three Zn^2+^ ions with high affinity. The truncation of HMA2c decreased the ATPase activity of AtHMA2, but enabled almost total complementation of the zinc deficiency phenotype of *A. thaliana hma2hma4* plants, despite partial mislocalization of the protein in the pericycle, suggesting that HMA2c is not essential for function (Eren *et al.*, 2006; Wong *et al.*, 2009). The function of the HMA4 C-terminal extension (HMA4c) remains more elusive, and contradictory results have been reported. In comparison with AtHMA2c, AtHMA4c is longer (470 amino acids) and contains approximately two times more Asp, Glu, and Cys residues (including 13 di-Cys motifs) and an 11 His-stretch at the C-terminal extremity (Supplementary Fig. S1). Consistently, it was estimated to interact with more Zn^2+^ ions (10) compared with AtHMA2c (Baekgaard *et al.*, 2010). The functional importance of AtHMA4c was tested in complementation experiments in yeast. A C-terminal truncated form of AtHMA4 was able to rescue the zinc sensitivity of a yeast mutant (Mills *et al.*, 2005), whereas in contradiction a His-stretch-deleted version was not (Verret *et al.*, 2005). Moreover, gradual deletion of AtHMA4c revealed a progressive increase in the ability of the protein to confer zinc tolerance to sensitive yeast (Baekgaard *et al.*, 2010). This is in contrast to a simultaneous study showing that a C-terminal-truncated AtHMA4 protein failed to complement the *hma2hma4* zinc deficiency phenotype (Mills *et al.*, 2010). Similar discrepancies were observed when analysing the effect of the C-terminal extension truncation of *Oryza sativa* HMA2 and HMA3 upon expression in yeast or in plants (Satoh-Nagasawa *et al.*, 2012; Kumagai *et al.*, 2014). Note that these rice mutant proteins localized properly in onion epidermal cells (Satoh-Nagasawa *et al.*, 2012; Kumagai *et al.*, 2014).

Interestingly, the HMA4c might account for functional differences between *A. thaliana* and *A. halleri* HMA4 proteins. Indeed, compared with AtHMA4c, the AhHMA4c exhibits 11 di-Cys motifs and a 10 His residue His-stretch. The amino acid sequences of the AtHMA4c and AhHMA4c display 64% of identity only, in contrast with other parts of the proteins sharing 96% amino acid sequence identity (Supplementary Fig. S1). These differences might arise from directional positive selection that operated on AhHMA4c during the evolutionary history of *A. halleri* and directly contribute to zinc/cadmium hyperaccumulation (Mano and Innan, 2008; Hanikenne *et al.*, 2013).

Here, the function *in vivo* and Zn^2+^ binding properties *in vitro* of the C-terminal extensions of the AtHMA4 and AhHMA4 were compared to examine potential functional divergence. Moreover, to alleviate conflicting results reported in the literature, a series of mutations were introduced in both HMA4c allowing assessment of the functional relevance of the whole domain, and of the di-Cys motif and His-stretch. We show that the C-terminal extensions of both proteins share a similar function and that they control, at least in part, the intracellular localization. We further reveal the key contribution of the di-Cys motifs, but not the His-stretch, to high affinity Zn^2+^ binding and function *in planta*. Finally, Zn^2+^ binding studies *in vitro* suggest that HMA4c is both a strong zinc chelator and a zinc donor.

## Materials and methods

### Plant material, growth condition, and transformation

*Arabidopsis thaliana* L. Heynhold (accession Columbia, Col-0) and *hma2hma4* double mutant *A. thaliana* plants (Col-0 background) (Hussain *et al.*, 2004) were used in all experiments. Prior to transformation, plants were grown on soil supplied with 1 mM ZnSO_4_ in a short-day growth chamber (22 °C and 8 h day^−1^ photoperiod) for 7 weeks. Plants were then transferred to long days (16 h day^−1^ photoperiod) and supplied with 3 mM ZnSO_4_ for 5 weeks to allow flowering. The plants were transformed using *Agrobacterium tumefaciens* by floral dipping (Clough and Bent, 1998).

For phenotyping and metal accumulation analysis on soil, third generation (T3) homozygous transgenic seeds were germinated in short days on 1/2 Murashige and Skoog (MS) agar medium containing 1% sucrose. After 14 d, seedlings were transferred to soil (potting mix, Brill TYPical, Tonerde 1/100 l) watered with tap water and grown for 6 weeks in long days prior to imaging and sample harvesting. For metal accumulation analysis in hydroponic conditions, the 2-week-old seedlings were instead transferred to hydroponic trays (Araponics, Belgium; Tocquin *et al.*, 2003) with modified Hoagland medium (Talke *et al.*, 2006; Charlier *et al.*, 2015; Nouet *et al.*, 2015) containing 1 µM ZnSO_4_ (control condition) and grown for 3 weeks in short days. The treatment was then initiated: plants were grown in the presence of 0.2 µM ZnSO_4_ (Nouet *et al.*, 2015). Nutrient solutions were changed weekly. After 3 weeks of treatment, root and shoot samples were harvested separately before processing for inductively coupled plasma atomic emission spectroscopy (ICP-AES) analyses or RNA extraction.

### Cloning

To generate the *pAtHMA4::AtHMA4* cassette, the full length coding sequence of the *A. thaliana HMA4* (*AtHMA4*) was cloned into the *pAtHMA4::AhHMA4* pBluescript II KS+ vector (pBKS) (Laurent *et al.*, 2016) in replacement of the full length coding sequence of the *A. halleri HMA4* (*AhHMA4*). The *pAtHMA4::AtAhHMA4* cassette was obtained by replacing in the same vector a fragment encoding residues 1–702 of AhHMA4 by the corresponding *AtHMA4* fragment using the In-Fusion HD cloning kit (Takara). The *pAtHMA4::AhAtHMA4* cassette was obtained by replacing in the *pAtHMA4::AtHMA4* pBKS a fragment encoding residues 1–702 of AtHMA4 by the corresponding *AhHMA4* fragment using the In-Fusion HD cloning kit. Synthetic genes encoding C-terminal fragments of AtHMA4 (residues 742–1172) with 11 His-stretch→11 Ala-stretch (HA variant) and 13 di-Cys→13 di-Ala motifs (CCAA variant) were obtained from GenScript (USA). The fragments were subsequently cloned into *pAtHMA4::AtHMA4* pBKS, using *Hpa*I/*Pac*I restriction sites to generate *pAtHMA4::AtHMA4HA* pBKS and *pAtHMA4::AtHMA4CCAA* pBKS, respectively. A fragment of the *pAtHMA4::AtHMA4CCAA* cassette, encoding the 1–1156 residues, was then cloned into *pAtHMA4::AtHMA4HA* pBKS in replacement of the corresponding region to obtain *pAtHMA4::AtHMA4CHA* pBKS (CHA variant, combining His-stretch and di-Cys mutations). For AhHMA4, synthetic genes encoding C-terminal fragments with 10 His-stretch→10 Ala-stretch (residues 1083–1163, HA variant) and 13 di-Cys→13 di-Ala motifs (residues 742–1163, CCAA variant) were obtained from GenScript. The fragments were subsequently cloned into *pAtHMA4::AhHMA4* pBKS (Laurent *et al.*, 2016), using *Spe*I/*Pac*I and *Hpa*I/*Pac*I restriction sites to generate *pAtHMA4::AhHMA4HA* pBKS and *pAtHMA4::AhHMA4CCAA* pBKS, respectively. A fragment of the *pAtHMA4::AhHMA4CCAA* cassette, encoding the residues 1–1148 was then cloned into *pAtHMA4::AhHMA4HA* pBKS in replacement of the corresponding region to obtain *pAtHMA4::AhHMA4CHA* pBKS (CHA variant). The *pAtHMA4::AhHMA4Ctrunc* cassette was obtained by cloning a fragment encoding the 1–702 residues of AhHMA4 directly followed by a stop codon into *pAtHMA4::AhHMA4* pBKS (Laurent *et al.*, 2016) in replacement of *AhHMA4*, using the In-Fusion HD cloning kit. To create binary vectors by plant transformation, all *promoter::cDNA* cassettes were finally cloned in a promoter-less variant of the pMDC32 vector (Curtis and Grossniklaus, 2003; Hanikenne *et al.*, 2008) after *Asc*I/*Pac*I-excision from the pBKS vectors. In contrast, a fragment encoding the AtHMA4 residues 1–702 was amplified by PCR with primers allowing the addition of an *Asc*I restriction site in 5′ and a stop codon followed by a *Pac*I restriction site in 3′ and subsequently cloned into the promoter-less variant of pMDC32 to generate *pAtHMA4::AtHMA4Ctrunc* pMDC32.

For localization experiments, the fragments encoding the AxHMA4CCAA, AxHMA4CHA, and AxHMA4Ctrunc variants were cloned into *pAhHMA4-2::AhHMA4::GFP* pBKS (Nouet *et al.*, 2015) in replacement of the *AhHMA4* coding sequence, using the In-Fusion HD cloning kit. All six *promoter::cDNA::GFP* cassettes were then cloned in a promoter-less variant of the pMDC32 vector (Curtis and Grossniklaus, 2003; Hanikenne *et al.*, 2008) after *Asc*I/*Pac*I-excision from pBKS. The *pAhHMA4-2::AtHMA4::GFP* pBKS vector used as control was already available from G. Lekeux *et al*., unpublished results. Note that the *pAhHMA4-2* promoter was preferred to the pAtHMA4 promoter as it supported higher expression levels facilitating green fluorescent protein (GFP) imaging (Nouet *et al.* 2015; Laurent *et al.* 2016).

For production in *E. coli*, synthetic genes with optimized codon usage encoding C-terminal fragments of AtHMA4 (residues 703–1172), the corresponding AtHMA4CCAA mutant, and AhHMA4 (residues 703–1163) were obtained from GenScript. The fragments were cloned into the pMalC2x vector (NEB), using *Eco*RI/*Hin*dIII restriction sites to allow expression in N-terminal fusion with maltose binding protein (MBP) generating the *MBP::AtHMA4c*, *MBP::AtHMA4CCAAc*, and *MBP::AhHMA4c* cassettes. Site-directed mutagenesis, using the QuikChange Site-Directed Mutagenesis method (Agilent Technologies) was then performed on the newly constructed *MBP::AtHMA4c* pMalC2x to insert a stop codon at the 3′ extremity of the sequence linking the sequence encoding MBP and AtHMA4c, allowing expression of the MBP protein alone.

All final constructions were verified by sequencing.

### Metal accumulation analyses

Shoot tissues were cleaned with milliQ water, while root tissues were desorbed as described (Talke *et al.*, 2006) and dried at 60 °C for 3 d. Shoot samples (10–50 mg of tissues) were then acid-digested in DigiPrep tubes with 3 ml of ≥65% HNO_3_ (Sigma-Aldrich) on a DigiPrep Graphite Block Digestion System (SCP Science) as follows: 15 min at 45 °C, 15 min at 65 °C, and 90 min at 105 °C. After cooling, sample volumes were adjusted to 10 ml with milliQ water, and 200 µl ≥65% HNO_3_ was added. Metal concentrations were determined using ICP-AES with a Vista-AX instrument (Varian, Melbourne, Australia) as described (Nouet *et al.*, 2015).

### Gene expression analyses

Total RNAs were extracted from root and shoot tissues separately using the RNeasy Plant Mini kit with on-column DNAse treatment (Qiagen). cDNAs were then synthesized from 1 µg of total RNA with the RevertAid H Minus First Strand cDNA Synthesis Kit (Thermo Fisher Scientific) using Oligo(dT). Quantitative RT-PCR reactions were performed in 384-well plates with a QuantStudio 5 Real-Time PCR system (Thermo Fisher Scientific) using Takyon™ Low Rox SYBR® MasterMix dTTP blue (Eurogentec). Three technical replicates were performed for each sample/primer pair (Supplementary Table S1). The reactions were performed in a total volume of 10 µl including 5 µl of Takyon™ Low Rox SYBR® MasterMix, 2.5 pmol of forward and reverse primers (Supplementary Table S1), and 4 µl of cDNA diluted 50×. The thermal profile used was: 2 min at 50 °C, 2 min at 95 °C, 40 repeats of 15 s at 95 °C, and 1 min at 60 °C, and a final stage of 15 s at 95 °C, 1 min at 60 °C, and 15 s at 95 °C to determine dissociation curves of the amplified products. The quality of the PCRs was checked visually through analysis of dissociation and amplification curves, and reaction efficiencies were determined for each PCR using the LinRegPCR software v2013 (Ruijter *et al.*, 2009). For each primer pair, mean reaction efficiencies were calculated from all reactions (Supplementary Table S1) and were then used to quantify relative gene expression levels by normalization using two reference genes, *At1g18050* and *EF1α* (Czechowski *et al.*, 2005), with the qBase software (Biogazelle; Hellemans *et al.*, 2007). The adequacy of the reference genes to normalize gene expression in the experimental conditions was checked using geNorm software in qBase (gene stability measure *M*=0.262, pairwise variation CV=0.091) (Vandesompele *et al.*, 2002).

### Confocal microscopy

T1 seeds of *A. thaliana* plants expressing the variant HMA4 proteins fused to GFP were germinated on 1/2 MS agar medium containing 1% sucrose and hygromycin B (20 µg ml^−1^) in short days. After 14 d, seedlings were transferred on the same medium without antibiotic. After 3 d, roots of three to six independent lines per construct were analysed. Images were collected at a 1024 × 1024-pixel resolution using a TCS SP5 inverted confocal laser microscope (Leica Microsystems) with a water-immersion PlanApochromat ×63 1.20 objective (Leica Microsystems) as previously described (Rausin *et al.*, 2010). An argon-ion laser (488 nm) was used for GFP excitation and the emission light was dispersed and recorded between 500 and 540 nm. Within one experiment, all images were acquired with the same excitation and detection settings (photomultiplier tube (PMT) gain, offset, ...) for all genotypes, with a PMT gain ensuring detection of GFP fluorescence only and excluding autofluoresence. To estimate HMA4 protein expression levels in root cells (Tillemans *et al.* 2005; Dubeaux *et al.* 2018), GFP fluorescence intensities were measured from confocal microscope images using ImageJ (https://imagej.nih.gov/ij/; accessed Sept 03, 2018) and plot profile analysis. Briefly, in pericycle cells expressing HMA4 fused to GFP, 10 optical sections were drawn across the transverse plasma membranes. GFP fluorescence intensity values (*n*=20) were then used to calculate a mean fluorescence intensity for each independent mutant line.

### Protein production and purification

*Escherichia coli* cells [strain BL21 (DE3)] transformed with the *MBP::AtHMA4c* pMalC2x, *MBP::AtHMA4CCAAc* pMalC2x, or *MBP::AhHMA4c* pMalC2x expression vector were grown at 37 °C in 500 ml terrific broth (TB) medium containing 50 µM ZnCl_2_, 100 µg ml^−1^ ampicillin and 2 g l^−1^ glucose. At an OD_600_ of ~0.6, the production was directly induced with 1 mM isopropyl β-D-thiogalactopyranoside. The culture was then incubated for 18 h at 18 °C. The cells, collected by centrifugation, were resuspended in 100 ml of 20 mM Tris/HCl (pH 7.5) with 200 mM NaCl, 2.5 mM tris(2-carboxyethyl)phosphine (TCEP) and 50 µM ZnCl_2_. A protease inhibitor cocktail (mini complete EDTA-free, Roche) and benzonase (Merck) were added. Cells were subsequently lysed using an EmulsiFlex-C3 cell disrupter (Avestin). The cellular extracts were clarified by centrifugation at 48000 *g* for 40 min at 4 °C. The soluble fraction was then loaded onto a 5 ml amylose resin high flow column (NEB) equilibrated in 20 mM Tris/HCl (pH 7.5) with 200 mM NaCl, 2.5 mM TCEP, 50 µM ZnCl_2_, and 1 mM phenylmethylsulfonyl fluoride (buffer A). The bound proteins were eluted with buffer A in the presence of 10 mM maltose. The fractions containing AtHMA4c, AtHMA4CCAAc, or AhHMA4c were pooled and directly loaded onto a HisTrap 5 ml column (GE Healthcare) equilibrated in buffer A. The bound proteins were washed with buffer A in the presence of 50 mM imidazole. They were then eluted in buffer A in the presence of 500 mM imidazole. The fractions containing AtHMA4c, AtHMA4CCAAc, or AhHMA4c were pooled and dialysed against 20 mM Tris/HCl (pH 7.5) with 200 mM NaCl, 1 mM TCEP and 100 µM ZnCl_2_. The MBP was similarly expressed in *E. coli* and purified on the amylose column only before being dialysed. The protein purity was assessed by SDS/PAGE.

### Zinc binding assay

To ensure metal removal and reductive conditions, proteins were incubated with an excess EDTA and TCEP for 3 h, followed by desalting in 50 mM MOPS (pH 7.3) with 100 mM NaCl, under anaerobic condition in a glovebox ([O_2_]<2 ppm). The protein concentration was estimated via a thiol assay with Ellman’s reagent, 5,5-dithiobis(2-nitrobenzoic acid), which reacts quantitatively with free cysteine thiols releasing the chromophore 5-mercapto-2-nitrobenzoate (λ_max_=418 nm; extinction coefficient ε=13600 M^−1^ cm^−1^) (Zimmermann *et al.*, 2009). This concentration estimation was warranted by (i) high detection sensitivity due to the high Cys contents in each protein target, (ii) unchanged Cys contents with/without addition of EDTA/SDS, demonstrating that there was neither metal-blocking of Cys thiols nor inaccessible thiols, and (iii) identical Zn^2+^ binding in the presence and absence of TCEP, demonstrating the absence of oxidized disulfide bonds.

Zn^2+^ binding assays were performed in 50 mM Mops (pH 7.3) with 100 mM NaCl and 1 mM TCEP; 1.0 µM AtHMA4c, AtHMA4CCAAc, or AhHMA4c and 22 or 111 µM of the zinc binding chromophoric ligand 4-(2-pyridylazo)resorcinol (Par) were used. Zn^2+^ and Par working solutions were prepared as described in Zimmermann *et al.* (2009). UV-visible spectra were recorded on a Varian Cary 300 spectrophotometer in dual-beam mode with quartz cuvettes with a path length of 1 cm.

The experiment was conducted based on following several relationships:

$$\overset{n}{\underset{i=1}{\sum }}i{\text{Zn}}^{\text{II}}{\left(\text{Par}\right)}_{2}+\;n\text{P}\;\;⇌\;\;\overset{n}{\underset{i=1}{\sum }}{\text{Zn}}_{i}^{\text{II}}\text{P}+\overset{n}{\underset{i=1}{\sum }}2i\text{Par}\;\;\;$$ (1)

$$\overset{n}{\underset{i=1}{\sum }}i{\text{Zn}}_{i}^{\text{II}}\text{P}=\;{\left[\text{Zn}\right]}_{\text{tot}}-[{\text{ Zn}}^{\text{II}}{\left(\text{Par}\right)}_{2}]$$ (2)

$$\bar{n}=(\overset{n}{\underset{i=1}{\sum }}i{\text{Zn}}_{i}^{\text{II}}\text{P})/{\left[\text{P}\right]}_{\text{tot}}$$ (3)

$${\text{Zn}}^{2+}+2\text{Par}⇌{\text{ Zn}}^{\text{II}}{\left(\text{Par}\right)}_{2}$$ (4)

$$\beta_{2}^{'}=\;\frac{\;[{\text{Zn}}^{\text{II}}{\left(\text{Par}\right)}_{2}]}{\left[{\text{Zn}}^{2+}\right]{\left[\text{Par}\right]}^{2}};\;\;\;\left[{\text{Zn}}^{2+}\right]=\frac{\;[{\text{Zn}}^{\text{II}}{\left(\text{Par}\right)}_{2}]}{\beta_{2}^{\text{'}}\;{\left[\text{Par}\right]}^{2}}$$ (5)

$${\text{Zn}}_{i}^{\text{II}}\text{P}\;⇌{\text{Zn}}_{i-1}^{\text{II}}\text{P}+{\text{Zn}}^{2+}$$ (6)

$$K_{\text{D}(i)}=\frac{\left[{\text{Zn}}_{i-1}^{\text{II}}\text{P}\right]\left[{\text{Zn}}^{2+}\right]}{\left[{\text{Zn}}_{i}^{\text{II}}\text{P}\right]}$$ (7)

Titration of a limited amount of Zn^2+^ into a solution containing the metal indicator Par and purified protein P (i.e. AtHMA4c, AtHMA4CCAAc, AhHMA4c, or MBP) induces competitive binding for the added Zn^2+^ ions between the Par ligand and the protein P according to an overall equilibrium described by Eq. (1). The Cys/His-rich C-terminal extension contains multiple Zn^2+^-binding sites with different affinities and can bind various numbers of Zn^2+^ ions. Consequently, the Zn^2+^-containing HMA4c species are a mixture of Zn^II^_*i*_-HMA4c (*i*=1, 2, … *n*) with the distribution of the number *i* depending on the Zn^2+^ availability. The total Zn^2+^ bound in $\overset{n}{\underset{i=1}{\sum }}i{\text{Zn}}_{i}^{\text{II}}\text{P}$ is equal to the difference between total Zn^2+^ added to the system and total Zn^2+^ bound by the Par metal indicator according to Eq. (2), and then the average number of zinc ions bound by the protein P under a specific condition is estimated by Eq. (3).

The Zn^2+^ availability in the system is estimated from the chromophoric response of Par upon Zn^2+^ binding according to Eqs (4) and (5) while Zn^2+^ binding to various protein sites may be described by Eqs (6) and (7). Since in the same experimental solution, the free Zn^2+^ concentration in Eqs (4) and (5) is identical to the free Zn^2+^ concentration in Eqs (6) and (7), the Zn^2+^ binding *K*_D_ defined by Eqs (6) and (7) can be monitored and estimated by the Par probe from the free [Zn^2+^] estimated from Eqs (4) and (5). The effective formation constants β′_2_ (=1/*K*_D_^eff^) for the complex Zn^II^(Par)_2_ have been estimated at a range of pH values where logβ′_2_=12.02 at pH 7.3 (Kocyła *et al.*, 2015). The equilibrium concentration of Zn^II^(Par)_2_ in the system was estimated directly from the characteristic solution absorbance at 500 nm with a reported pH-dependent extinction coefficients that is 70.1 mM^−1^ cm^−1^ at pH 7.3 (Kocyła *et al.*, 2015). The total free [Par] that does not bind Zn^2+^ was obtained by the relationship [Par]=[Par]_tot_−2[Zn^II^(Par)_2_] and free [Zn^2+^] by Eq. (5).

Stepwise titration of Zn^2+^ into the solution containing Par and HMA4c will increase free [Zn^2+^] concentration in the solution and this will in turn increase the Zn^2+^ occupations on HMA4c [Eq. (3)] and/or Par [Eq. (4); detected by A_500_], depending not only on the affinity of HMA4c for Zn^2+^, but also on the total Par concentration in the solution. These relationships may be analysed graphically by plotting an average Zn^2+^ occupation number n [defined by Eq. (3)] on HMA4c versus Zn^II^(HPar)_2_ formation (via *A*_500_) and log[Zn^2+^] [via Eq. (5)], as shown in Fig. 6.

## Results

### Function of the HMA4 C-terminal extension *in vivo*

**Fig. 1.**
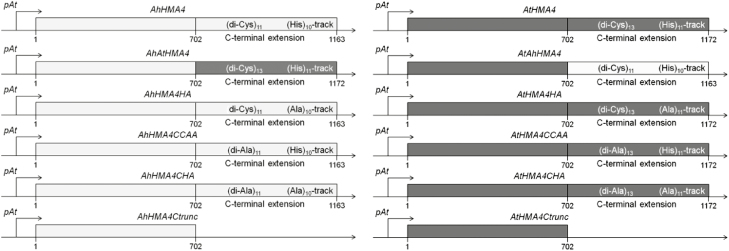
Overview of the HMA4 variant constructs for complementation experiments in plants. The expression cassettes are schematically represented. The nucleotide sequences encoding *A. thaliana* (dark gray) or *A. halleri* (light gray) native or mutant HMA4 proteins are preceded by the *A. thaliana HMA4* promoter (pAt, 2595 bp). Numbers correspond to the amino acid position in the HMA4 protein. Ah, *A. halleri*; At, *A. thaliana*; AtAhHMA4 and AhAtHMA4, swapped C-terminal extensions; HA: His- → Ala-stretch; CCAA: di-Cys → di-Ala motifs; CHA: HA and CCAA mutations combined; Ctrunc: fully truncated C-terminal extension.

To assess and compare the function of the C-terminal domains of the *A. thaliana* and *A. halleri* HMA4 proteins, a series of mutants were generated as summarized in Fig. 1. First, chimeras consisting of the sequence coding for the AtHMA4 N-terminal and TM region followed by the coding sequence of AhHMA4c (AtAhHMA4) and vice versa (AhAtHMA4) were generated to assess whether the C-terminal extension is responsible for functional differences between the two proteins. Second, to tackle the specific importance of the HMA4c di-Cys motifs and His-stretch separately, they were respectively replaced by di-Ala motifs and a poly-Ala-stretch to create respectively the AxHMA4CCAA and AxHMA4HA mutant proteins (with x being t for *thaliana* or h for *halleri*). Third, these mutations were combined in the AxHMA4CHA proteins. Finally, the importance of the C-terminal extensions themselves was reassessed, truncating the C-terminal extension of both proteins (AxHMA4Ctrunc). Native and mutant forms of both genes were expressed under the control of the endogenous *AtHMA4* promoter (*pAtHMA4*) in the loss-of-function *hma2hma4 A. thaliana* mutant (Hussain *et al.*, 2004). Four to eight independent homozygous lines (T3 generation) were obtained for each construct.

**Fig. 2.**
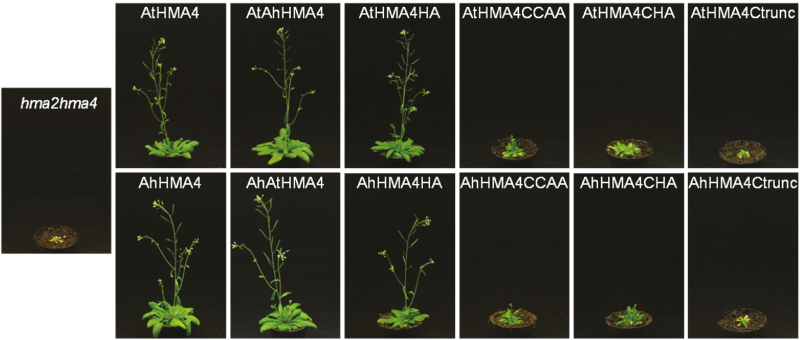
Complementation of the *A. thaliana hma2hma4* zinc deficiency phenotype. HMA4 variants were expressed in *hma2hma4* plants under the control of the *AtHMA4* promoter. The plant phenotypes are shown after 6 weeks of growth on soil without zinc supplementation. Non-transformed *hma2hma4* plants or expressing the native HMA4 proteins were respectively used as negative and positive controls. Images are representative of multiple observations of four to eight independent homozygous T3 lines for each genotype. Ah, *A. halleri*; At, *A. thaliana*; AtAhHMA4 and AhAtHMA4, swapped C-terminal extensions; HA: His- → Ala-stretch; CCAA: di-Cys → di-Ala motifs; CHA: HA and CCAA mutations combined; Ctrunc: fully truncated C-terminal extension.

Both native AxHMA4 proteins complemented the *hma2hma4* phenotype and allowed plants to develop normally until seed setting, when grown on soil watered with tap water (Fig. 2). The *hma2hma4* plants expressing the AtAhHMA4, AhAtHMA4, and AxHMA4HA variants grew similarly to those expressing the native proteins. In contrast, the expression of the AxHMA4Ctrunc variants completely failed in complementing the zinc deficiency phenotype of the *hma2hma4* mutant, as reported previously for AtHMA4 (Mills *et al.*, 2010). Indeed, plants expressing these C-truncation variants exhibited a stunted growth and chlorotic aspect identical to the *hma2hma4* mutant plants (Fig. 2). An intermediate complementation was observed in plants expressing the AxHMA4CCAA and AxHMA4CHA variants. Although their growth was strongly impaired, with chlorosis and a bushy aspect, they were not as dramatically affected as the *hma2hma4* mutant. Some of them were able to flower, yet they could not set functional seeds (Fig. 2).

**Fig. 3.**
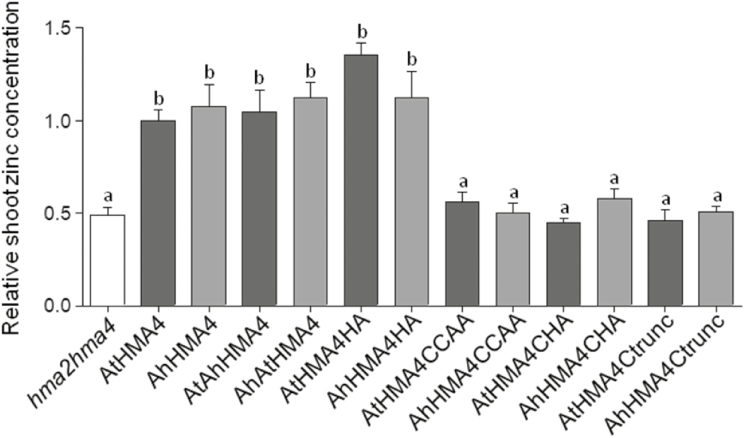
Zinc accumulation in complemented plants grown on soil. Non-transformed *hma2hma4* mutant (white) and expressing the native or mutant *A. thaliana* (dark gray) or *A. halleri* (light gray) HMA4 proteins under the control of the *AtHMA4* promoter were grown for 6 weeks on soil without zinc supplementation. Zinc concentrations were measured in shoot tissues collected from two plants per line. Values are relative to lines expressing the native AtHMA4 proteins and are means±SEM of four to eight homozygous T3 independent lines for each genotype. The data were analysed with one-way ANOVA followed by Tukey’s multiple comparison test. Statistically significant differences (*P*<0.05) between means are indicated by different letters. Ah, *A. halleri*; At, *A. thaliana*; AtAhHMA4 and AhAtHMA4, swapped C-terminal extensions; HA: His- → Ala-stretch; CCAA: di-Cys → di-Ala motifs; CHA: HA and CCAA mutations combined; Ctrunc: fully truncated C-terminal extension.

Zinc accumulation in rosette leaves was then determined for all genotypes grown on soil watered with tap water. Expression of the native proteins increased shoot zinc accumulation by 2-fold compared with the *hma2hma4* mutant. No differences were observed between the ability of AtHMA4 and AhHMA4 to increase shoot zinc levels (Fig. 3). Consistent with their restored visual phenotype, plants expressing the AtAhHMA4, AhAtHMA4, and AxHMA4HA variants displayed zinc levels in shoots similar to those expressing the native proteins. In contrast, plants expressing the remaining variants (AxHMA4CCAA, AxHMA4CHA, and AxHMA4Ctrunc) exhibited shoot zinc contents identical to the *hma2hma4* mutant, again in agreement with their visual phenotype (Fig. 3).

**Fig. 4.**
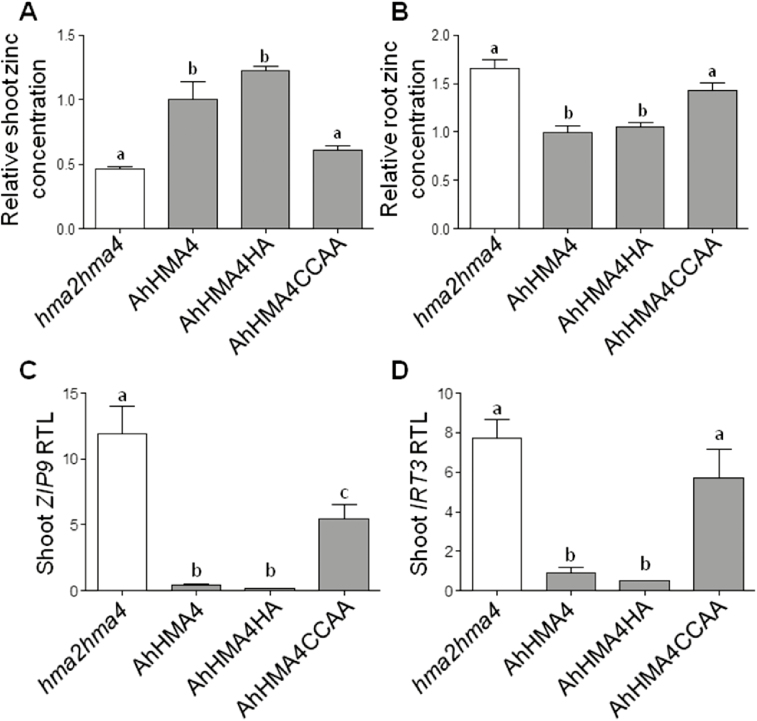
Zinc accumulation and expression of zinc deficiency response genes in complemented plants grown in hydroponic conditions. Non-transformed *hma2hma4* mutant (white) and expressing the native or mutant *A. halleri* HMA4 proteins (medium gray) under the control of *AtHMA4* promoter were grown for the last 3 weeks before harvest in Hoagland hydroponic medium containing 0.2 µM zinc. Zinc concentrations were measured in shoot (A) and root (B) tissues collected from two plants per line. Values are relative to lines expressing the native AhHMA4 proteins and are means±SEM of two independent lines from three biological replicates. Transcript levels were quantified from plant tissues collected from two plants per line. Relative transcript levels (RTL) of *IRT3* (C) and *ZIP9* (D) in shoots are mean±SEM of two independent lines from two biological replicates. The data were analysed with one-way ANOVA followed by Tukey’s multiple comparison test. Statistically significant differences (*P*<0.05) between means within each figure panel are indicated by different letters. Ah: *A. halleri*; HA: His-stretch→Ala-stretch; CCAA: di-Cys→di-Ala motifs.

Zinc distribution in plant tissues was further detailed for a subset of constructs (AhHMA4, AhHMA4HA, and AhHMA4CCAA) upon growth in hydroponic medium (0.2 µM zinc) (Nouet *et al.*, 2015). Plants expressing the native protein accumulated respectively about 2-fold higher and 1.5-fold lower zinc levels in shoots and roots compared with the *hma2hma4* mutant, respectively (Fig. 4A, B). In agreement with the soil experiment, AhHMA4HA plants displayed zinc levels similar to AhHMA4 plants in both roots and shoots. Finally, zinc levels in AhHMA4CCAA plants were similar to levels found in the *hma2hma4* mutant with, although not significant, slightly higher and lower zinc levels in shoots and roots compared with the mutant, respectively (Fig. 4A, B). Note that no other major changes were observed in the ionome of the plants (Supplementary Table S2). As with the *hma2hma4* mutant, the AhHMA4CCAA plants, however, displayed slightly lower manganese, calcium, and magnesium levels in shoots compared with complemented plants (AhHMA4 and AhHMA4HA).

To further assess the zinc status in the plants, the expression levels of *ZIP9* and *IRT3*, genes whose expression is induced upon zinc deficiency and repressed upon zinc excess (Talke *et al.*, 2006), were assessed in shoot tissues, which strongly respond to zinc deficiency in the *hma2hma4* mutant (Nouet *et al.*, 2015). The *IRT3* and *ZIP9* shoot transcript levels were respectively about 8- and 30-fold higher in the *hma2hma4* mutant than in AhHMA4 and AhHMA4HA plants. In contrast, *ZIP9*, and although not significantly *IRT3*, transcript levels in AhHMA4CCAA shoots were intermediate between the *hma2hma4* mutant and AhHMA4 or AhHMA4HA plants (Fig. 4C, D). Thus, both shoot and root zinc accumulation as well as shoot expression of zinc status marker genes displayed subtle differences between the *hma2hma4* and AhHMA4CCAA plants (Fig. 4), possibly explaining the slightly improved growth of AhHMA4CCAA plants compared with the *hma2hma4* mutant (Fig. 2).

### Function of the HMA4 C-terminal extension in protein localization

**Fig. 5.**
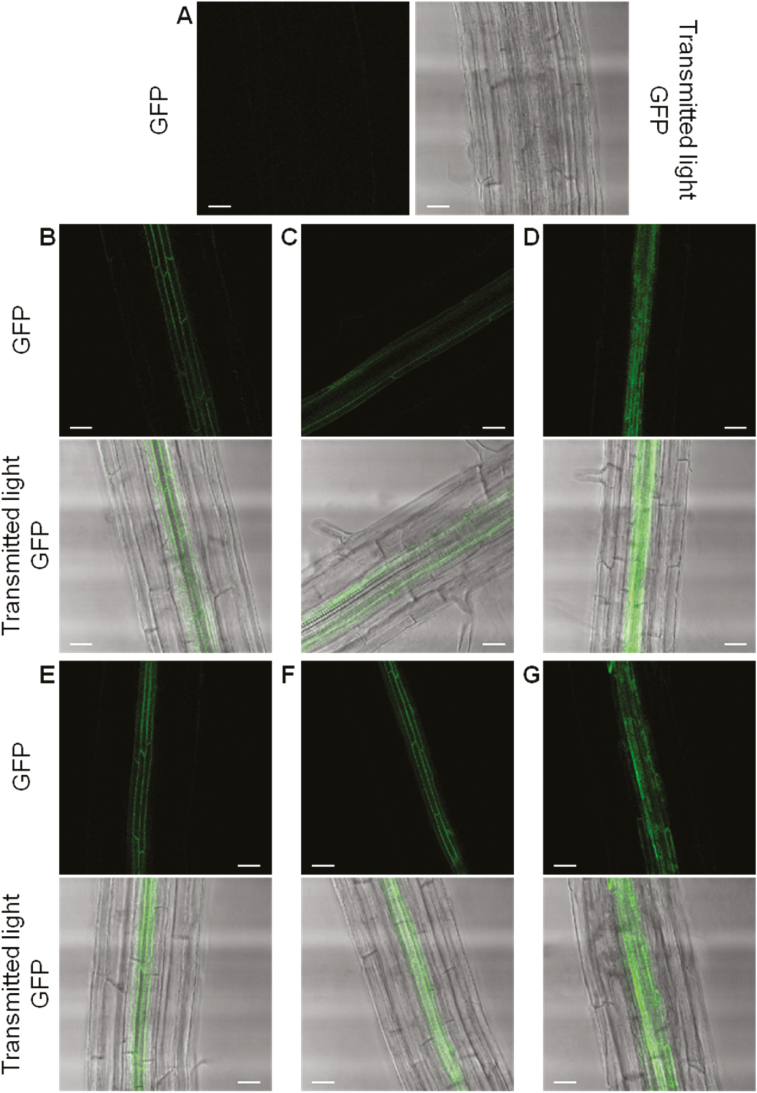
HMA4 variant localization in *A. thaliana*. The AtHMA4CCAA (B), AtHMA4CHA (C), AtHMA4Ctrunc (D), AhHMA4CCAA (E), AhHMA4CHA (F), and AhHMA4Ctrunc (G) variants fused to GFP and expressed under control of the copy 2 *AhHMA4* promoter in Col-0 were imaged by confocal microscopy in roots of 18-day-old T1 seedlings. Non-transformed Col-0 seedlings (A) were used as negative controls. The images are, for each genotype, representative of three to six independent lines. Scale bars 25 µm. Ah, *A. halleri*; At, *A. thaliana*; CCAA: di-Cys→di-Ala motifs; CHA: HA: His- → Ala-stretch; CCAA: di-Cys → di-Ala motifs; CHA: HA and CCAA mutations combined; Ctrunc: fully truncated C-terminal extension.

Whether the localization and/or the protein expression level of the variants unable to complement the *hma2hma4* mutant were altered was next examined. The AxHMA4CCAA, AxHMA4CHA, and AxHMA4Ctrunc variants were expressed in fusion to GFP, under the control of the *A. halleri HMA4 promoter 2* (*pAhHMA4-2*) in the Col-0 genetic background (Nouet *et al.*, 2015). Roots of 18-day-old seedlings were examined by confocal microscopy. Non-transformed *hma2hma4* seedlings did not emit any fluorescence (Fig. 5A). The native AtHMA4 and AhHM4 proteins were previously shown to localize to the plasma membrane of root pericycle cells in *A. thaliana* when expressed under the control of *pAhHMA4-2* (Nouet *et al.*, 2015; G. Lekeux *et al*., unpublished results), which was also observed for the AxHMA4CCAA and AxHMA4CHA, with no evidence of GFP aggregation in cells (Fig. 5B, C, E, F). Protein expression levels in cells were estimated for the four variants through GFP quantification and were similar to the expression level of the native AtHMA4 protein (Supplementary Fig. S2). The inability of the AxHMA4CCAA and AxHMA4CHA variants to complement the *hma2hma4* phenotype therefore likely results from an impaired function of the protein in plants. In contrast, the AxHMA4Ctrunc variants were also detected in root pericycle cells, yet the GFP signal in the plasma membrane was not well defined and a high proportion of diffuse and dotted signal was detected inside the cells, suggesting protein retention in the membrane of an intracellular organelle (Fig. 5D, G; Supplementary Fig. S3). This altered localization likely accounted for the inability of the Ctrunc variants to complement the *hma2hma4* mutant.

### Quantification of Zn^2+^ binding properties of isolated native C-terminal sequences and a CCAA variant

To provide molecular insights into the observed consequences of replacement of the C-terminal di-Cys motifs by di-Ala motifs on the HMA4 functions *in vivo*, the native AxHMA4 C-terminal extensions (AxHMA4c) and a corresponding protein variant, AtHMA4CCAAc, were expressed in *E. coli* and purified for zinc binding assay *in vitro*. To overcome a protein solubility problem and to facilitate the protein purification, the nucleotide sequences encoding AxHMA4c and AtHMA4CCAAc were fused to the C-terminal gene sequence of a maltose binding protein (MBP) and the target proteins expressed and purified as MBP-fusion proteins. The zinc binding assay was conducted by titrations of the purified protein samples with Zn^2+^ in the presence of a zinc binding chromophoric ligand, 4-(2-pyridylazo)resorcinol (Par) (Zimmermann *et al.*, 2009; Kocyła *et al.*, 2015). Here the ligand Par acts as a metal buffer controlling free Zn^2+^ concentrations in solution and as a metal speciation indicator monitoring zinc binding to the protein under various conditions (see ‘Materials and methods’ section for details).

**Fig. 6.**
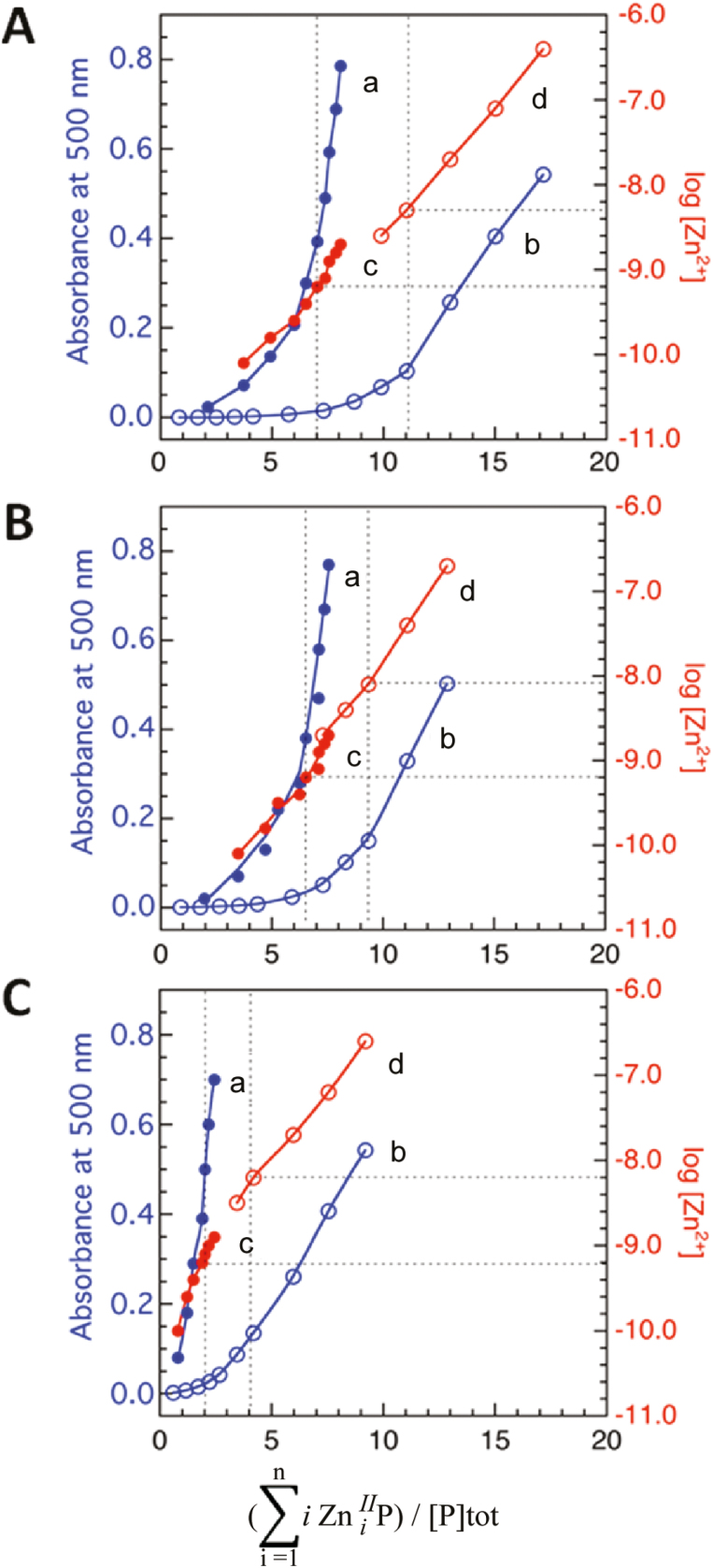
Quantification of Zn^2+^ binding affinity and stoichiometry of isolated proteins AtHMA4c (A), AhHMA4c (B), and AtHMA4CCAAc (C) with Par probe at either 22 µM (open circles) or 111 µM (solid circles). (a, b) Variation of A_500_ with average zinc binding stoichiometry of proteins; (c, d) Variation of log [Zn^2+^] with average zinc binding stoichiometry of proteins. The experiments were conducted by titration of each protein (1.0 µM) in MOPS buffer (50 mM, pH 7.3) containing NaCl (100 mM) and TCEP (1 mM) in the presence of excess Par ligand.

Titration of Zn^2+^ into a MOPS buffer (pH 7.3) containing Par (111 µM) and AtHMA4c (1.0 µM) allowed monitoring of Zn^2+^ binding to the protein at free Zn^2+^ concentrations in the subnanomolar range (Fig. 6A, c). In such a solution, AtHMA4c was detected to bind about 7 equiv of zinc with dissociation constants *K*_D_<1 nM (Fig. 6A, a); see Eqs 6 and 7 in the ‘Materials and methods’ section for the definition of *K*_D_ and its relationship to the free Zn^2+^ concentrations in solution). Equivalent titration but with lower total Par concentration (22 µM) increased the sensitive detection range of zinc binding to protein by ~1000-fold to free Zn^2+^ concentrations <1 µM (Fig. 6A, d). This titration confirmed the above-mentioned subnanomolar affinity zinc binding sites for the seven equivalents of zinc and detected further binding of ~4 equivalents of Zn^2+^ with *K*_D_ in the range of 1–10 nM and of more than six equivalents of weak zinc binding with *K*_D_ in the range of 10–1000 nM (Fig. 6A, b). However, such weak Zn^2+^ binding at *K*_D_>10 nM might be of little biological significance in nature. Control experiments carried out in the same conditions confirmed that the MBP carrier protein alone did not compete with Par for Zn^2+^, and therefore lacked Zn^2+^ binding sites.

Likewise, the native sequence of AhHMA4c was detected to possess comparable Zn^2+^-binding capacity under the same conditions: ~6 equivalents of zinc with *K*_D_<1 nM, ~3 equivalents with *K*_D_=1–10 nM and >3 equivalents with *K*_D_=10–1000 nM, but the overall Zn^2+^ binding number was reduced (Fig. 6B). This may be attributed to the reduced number of di-Cys motifs in AhHMA4c (Supplementary Fig. S1).

In contrast, the equivalent experiments on the protein variant AtHMA4CCAAc detected that the protein can bind ~2 equivalents of zinc with *K*_D_<1 nM and another two with *K*_D_=1–10 nM and more than five with *K*_D_=10–1000 nM (Fig. 6C). This demonstrated that a replacement of the 13 di-Cys motifs by the di-Ala motifs compromised the high affinity Zn^2+^ binding sites (with *K*_D_<10 nM) specifically. The variant retained comparable capacity for weak Zn^2+^ binding (*K*_D_>10 nM), which, again, might be of little biological significance.

## Discussion

This comparative analysis of HMA4 variant proteins allowed a deeper understanding of the function of the C-terminal extension of the protein, and provides plausible interpretations for conflicting results from previous reports on this function (see below). The study highlighted the particular importance of the di-Cys motifs. It further emphasized that studying a protein *in vivo* in its endogenous context is key for proper functional characterization. Here, the *A. thaliana* HMA4 promoter was used to express HMA4 variants in the *hma2hma4* mutant background. In addition, point mutations replacing native residues by Ala residues rather than truncations was favored to address the importance of specific amino acids.

### The His-stretch in the HMA4 C-terminal extension is not required for function

In this study, replacing the AxHMA4 His-stretch by an Ala-stretch (Fig. 1) did not impair the ability of the protein to complement the *A. thaliana hma2hma4* zinc deficiency phenotype. The visual phenotype, and root and shoot zinc accumulation, as well as the expression of zinc deficiency marker genes, were restored (Figs 2–4), suggesting that the His-stretch is not essential for the protein function *in vivo*. In contrast, truncating the *A. thaliana* HMA4 C-terminal extremity to delete the His-stretch was shown to disable the ability of the protein to rescue the zinc sensitivity of a mutant yeast (Verret *et al.*, 2005). This contradiction suggests that one of the amino acid residues downstream of the His-stretch might be important for the protein function. Interestingly, a Ser residue is present in the penultimate position of the HMA4 protein. As reported for the penultimate Thr residue of the H^+^-ATPase C-terminal extension, the phosphorylation of this Ser residue might regulate the protein function (Fuglsang *et al.*, 1999; Svennelid *et al.*, 1999; Ottmann *et al.*, 2007; Ekberg *et al.*, 2010).

### Deleting the HMA4 C-terminal extension results in altered localization and loss of function

Conflicting results were also reported regarding the impact of C-terminal truncations of *A. thaliana* HMA4, depending on the complementation assay set-up (yeast or *A. thaliana*). An HMA4 version with fully truncated C-terminal extension was still able to complement zinc sensitivity of a mutant yeast (Mills *et al.*, 2005). Moreover, sequential deletion of the di-Cys motifs of HMA4 revealed a progressive increase in zinc tolerance when the deleted versions were expressed in a zinc sensitive yeast strain (Baekgaard *et al.*, 2010). On the other hand, full truncation of the HMA4 C-terminal extension led to a complete lack of complementation when the protein was expressed in *A. thaliana hma2hma4* plants (Mills *et al.*, 2010). However, in the latter study, this observation may stem from ectopic and constitutive expression resulting in protein overexpression and mislocalization. In the present study, an identical phenotype was observed when the Ctrunc form of AxHMA4 was expressed under the control of the endogenous *pAtHMA4* in the *hma2hm4* mutant (Figs 2, 3), and the truncated proteins showed altered localization (Fig. 5D, G; Supplementary Fig. S3). The C-terminal truncation might trigger the retention of the protein in the endoplasmic reticulum, as the protein signal roughly delineated a membrane system and surrounded the nucleus (Supplementary Fig. S3) (Baekgaard *et al.*, 2010). This mislocalization of the HMA4 version with fully truncated C-terminal extension likely accounts for its inability to complement the *hma2hma4* in Mills *et al.* (2010) as well as in our study and might also explain the discrepancy with the results obtained in yeast (Mills *et al.*, 2005; Baekgaard *et al.*, 2010). It also prevents determination of the function of HMA4c as a whole, as the effect on function of (i) the mis-localization and (ii) the lack of amino acid residues required for metal binding can be confounded. However, we show here the key contribution of di-motifs of HMA4c (see below for discussion).

The truncation of the *A. thaliana* HMA2c also led to protein mislocalization, with a diffuse distribution in the cytoplasm (Wong *et al.*, 2009). In contrast, the truncation of the HMA2 N-terminal domain, mutations in the CCTSE motif in the N-terminal domain of HMA4, as well as mutation of the residues forming the zinc permeation pathway of HMA4 did not alter their intracellular localization (Wong *et al.*, 2009; Laurent *et al.*, 2016; G. Lekeux *et al*., unpublished results). It is thus tempting to hypothesize that the signal targeting HMA4 to the plasma membrane might stand in the C-terminal extension. In particular, di-Leu motifs, whose mutations were previously shown to alter membrane protein trafficking, are present in the HMA4 C-terminal extension (Petris *et al.*, 1998; Petris and Mercer, 1999; Wang *et al.*, 2014*b*). In addition, HMA4c is rich in Ser and Thr residues. The phosphorylation of such residues has been proposed to be related to the regulation of ATP7B cellular trafficking (Lutsenko, 2016). Alternatively, it is not excluded that the C-terminal truncation might disrupt proper protein folding and thereby cause its retention in the ER (Lefebvre *et al.*, 2012). Such misfolding might trigger protein aggregation, explaining the presence of GFP fluorescence clusters in cells expressing the C-terminal-truncated AxHMA4 protein (Fig. 5D, G; Supplementary Fig. S3). However, the C-terminal extension might not be as important for the localization of some other HMA proteins, as the truncation of OsHMA2 and OsHMA3 C-terminal extensions did not alter their localization in onion epidermal cells (Satoh-Nagasawa *et al.*, 2012; Kumagai *et al.*, 2014). Note that the C-terminal extensions of these proteins do not exhibit any di-Leu motifs.

### The di-Cys motifs in the HMA4 C-terminal extension are required for function and confer nanomolar affinity for multiple zinc ions

To assess their function, all di-Cys motifs present in the C-terminal extension of AxHMA4 were replaced by di-Ala motifs (Fig. 1). The expression of this variant partially complemented the zinc deficiency phenotype of the *hma2hma4* mutant (Figs 2–4), despite proper expression at the transcript and protein levels (Supplementary Figs S2, S4) and proper cellular localization (Fig. 5B, E; Supplementary Fig. S3), thus highlighting the key contribution of these di-Cys motifs to the HMA4 protein function. The same phenotype was observed when di-Cys→di-Ala and His-stretch→Ala-stretch mutations were combined (Figs 1–4), again confirming the low importance of the His-stretch (Figs 2–4).

Following production and purification in *E. coli*, the Zn^2+^ binding capability of both native AxHMA4c proteins and a selected variant, AtHMA4CCAAc, were examined *in vitro*. Both native proteins were shown to be able to bind multiple zinc ions (>10) with a continuous spectrum of different affinities from subnanomolar to micromolar, but it was not possible to define individual binding sites and their affinity (Fig. 6). High binding capacity was anticipitated from their protein sequences that are rich in putative metal-binding ligands (Cys, His, Asp, and Glu residues) including multiple di-Cys motifs (Supplementary Fig. S1). Nevertheless, we were able to detect that AtHMA4c can bind ~11 Zn^2+^ ions with *K*_D_<10 nM, in excellent agreement with a previous observation that an AtHMA4c protein form containing ~10 equivalents of Zn^2+^ could be isolated by size-exclusion chromatography (Baekgaard *et al.*, 2010). It is possible that only those Zn^2+^ ions bound by the protein with nanomolar affinity or below can survive the size-exclusion chromatography elution. It is also likely that only such high affinity Zn^2+^ binding sites are biologically significant.

AhHMA4c was shown here to possess comparable binding affinities for all types of Zn^2+^ binding but with some marginal decrease in binding capacity, i.e. lower number of bound Zn^2+^ ions. This is likely a consequence of a decreased number of di-Cys motifs in AhHMA4c relative to that in AtHMA4c (11 *vs* 13) although their total Cys numbers are comparable (42 *vs* 44) (Supplementary Fig. S1). Nevertheless, these differences did not account for functional differences, as AtHMA4 and AhHMA4 identically complemented the *hma2hma4* mutant (Figs 2–4), demonstrating that both C-terminal extensions possess more than enough Zn^2+^ binding capacity for their cellular functions.

In contrast, a replacement of all 13 di-Cys motifs by di-Ala in AtHMA4CCAAc reduced the number of high affinity Zn^2+^ binding sites specifically (from ~11 to ~4 with *K*_D_<10 nM) while the weak binding sites (*K*_D_>10 nM) were largely retained (see Fig. 6). A di-Cys motif in the N-terminus of AhHMA4 was recently shown to constitute a Zn^2+^ site with *K*_D_=6 nM, but a contribution of a nearby Glu as a co-ligand enhanced the affinity by 25-fold to a *K*_D_=0.25 nM (Laurent *et al.*, 2016). These experiments suggest that di-Cys motifs contribute dominantly to the high affinity Zn^2+^ binding sites but other co-ligands are required for the observed high Zn^2+^ binding affinity at subnanomolar levels. The severe functional impairment of both AxHMA4CCAA proteins in complementation of the *hma2hma4* mutant (Figs 2–4) highlights the functional importance of Zn^2+^ binding sites with *K*_D_<10 nM and the biological irrelevance of those weak sites with *K*_D_>10 nM. Notably, AtHMA4CCAAc still retained limit binding sites for four Zn^2+^ ions with *K*_D_<10 nM. These sites may be either contributed originally from other non-di-Cys ligands such as single Cys or numerous His, Asp, and Glu residues also present in HMA4c (Supplementary Fig. S1), or from structural coordination rearrangement induced upon removal of di-Cys motifs, creating new high affinity binding sites. Nevertheless, limited high affinity Zn^2+^ binding in AxHMA4CCAA is not sufficient for the proper cellular functions, but may account for the observed residual activity in complementing the function of the *hma2hma4* mutant (Figs 2–4).

In agreement with the putative importance of the C-terminal extension through high affinity Zn^2+^ binding, the truncation of *A. thaliana* HMA2c, which binds three Zn^2+^ with high affinity, was shown to intermediately decrease the activity of the protein *in vitro* (Eren *et al.*, 2006). A tendency towards partial or complete loss of function was also observed when truncating the HMA2 N-terminal extension (Wong *et al.*, 2009) or mutating the high affinity Zn^2+^ binding motif of both HMA2 and HMA4 N-terminal extensions (Eren *et al.*, 2007; Zimmermann *et al.*, 2009; Laurent *et al.*, 2016). This is, however, in contrast to the previous report showing that the progressive deletion of the di-Cys motifs gradually increased the ability to confer zinc resistance to a sensitive yeast (Baekgaard *et al.*, 2010). This contradiction might be explained by the lack of a plant component (such as a putative interactor) when the experiments are carried out in yeast.

The presence of multiple metal binding sites with different affinities is a common feature for Cys-rich proteins and may be important for scavenging metal ions with high affinity in metal-limiting conditions and to bind excess metal ions for export when the metal supply is too high. For example, Cys-rich human metallothionein possesses three types of Zn^2+^ binding sites with different affinities and can act as both a strong Zn^2+^ binder and a donor (Krężel and Maret, 2007, 2017). The Cys-rich C-terminus of HMA4 proteins may assume similar functions. Moreover, as has already been proposed for cytosolic extensions found in zinc/cadmium P_IB_ ATPases, HMA4c might regulate the protein activity, possibly by interacting with other domains of the protein (Mitra and Sharma, 2001; Eren *et al.*, 2006, 2007; Liu *et al.*, 2006; Wong *et al.*, 2009; Wang *et al.*, 2014*a*; Laurent *et al.*, 2016; G. Lekeux *et al*., unpublished results). A putative interaction with another cytosolic protein typical of plants should not be neglected too.

### Sequence divergence among AtHMA4c and AhHMA4c does not result in functional divergence

Finally, the *A. thaliana* and *A. halleri* HMA4 proteins, as well as chimeric proteins with swapped C-terminal extensions, were equally able to complement the *hma2hma4* mutant, restoring similar growth on soil and shoot zinc accumulation (Figs 2–3). The C-terminal domains of the two proteins displayed consistent Zn^2+^ binding properties *in vitro* (Fig. 6), whereas the CCAA and CHA mutations of the two proteins resulted in the same phenotypes when assessed *in vivo* (Figs 2, 3). High sequence divergence between these two domains does not seem to support essential functional differences. The contribution of HMA4 to higher zinc translocation from root to shoot in *A. halleri* compared with *A. thaliana* thus appears to exclusively result from higher expression of HMA4 in *A. halleri* (Talke *et al.*, 2006; Courbot *et al.*, 2007; Hanikenne *et al.*, 2008). At a biochemical level, it suggests a flexible organization of the C-terminal extensions of the AtHMA4 and AhHMA4 proteins, which despite a high sequence divergence, fulfill the same functions *in vivo*.

## Supplementary data

Supplementary data are available at *JXB* online.

Fig. S1. Amino acid sequence alignment of plant P_IB-2_ ATPase C-terminal extensions.

Fig. S2. HMA4 native and variant protein expression level in *A. thaliana*.

Fig. S3. Closer views of HMA4 variant localization in *A. thaliana*.

Fig. S4. *AhHMA4* native and variant gene expression levels in complemented plants grown in hydroponic conditions.

Table S1. Sequences and reaction efficiencies of quantitative RT-PCR primer pairs.

Table S2. Ionome profile of complemented plants grown in hydroponic conditions.

Supplementary Tables S1-S2 and Figures S1-S4
