# Supplementary material for: di-Cysteine motifs in the C-terminus of plant HMA4 proteins confer nanomolar affinity for zinc and are essential for HMA4 function in vivo
Source: J Exp Bot. 2018 Aug 22;69(22):5547–60. doi: 10.1093/jxb/ery311 (PMC6255694; doi:10.1093/jxb/ery311)
Supplement: Supplementary Tables S1-S2 and Figures S1-S4 [file ery311_suppl_supplementary_tables_s1-s2_figs_s1-s4.pdf]

Table S1. Sequences and reaction efficiencies of quantitative RT-PCR primer pairs.

Gene	Primer	Sequence 5'=>3'	Reaction efficiency	Standard deviation RE
At1g58050	Fwd	CCATTCTACTTTTTGGCGGCT	1.98	0.019
	Rev	TCAATGGTAACTGATCCACTCTGATG		
AtEF1α	Fwd	TGAGCACGCTCTTCTTGCTTTCA	1.943	0.021
	Rev	GGTGGTGGCATCCATCTTGTTACA		
AtZIP9	Fwd	CCATCACTACTCCGATCGGTGT	1.875	0.022
	Rev	CACCAATGCTGCAACGCTATAA		
AtIRT3	Fwd	AGTCATCCTCCTGGTCATGATT	1.968	0.019
	Rev	GAGCATGACCAATGTCGAT		
AtHMA4	Fwd	AGCTAGCTACAGGGCGACAGCA	1.93	0.014
	Rev	CAGCTTTAACCGCTACAACTGTGCT		

RE= reaction efficiency

Table S2. Ionome profile of complemented plants grown in hydroponic condition.

A. Shoot

Genotype	Zn		Cu		Fe		Mn		Ca		K		Mg	
	Mean	SEM	Mean	SEM	Mean	SEM	Mean	SEM	Mean	SEM	Mean	SEM	Mean	SEM
hma2hma4	15.2^{a}	0.6	16.3^{a}	0.7	78.0^{a}	3.3	236.7^{a}	6.4	54970.0^{a}	1302.6	39230.5^{a}	855.4	6713.3^{ad}	147.3
AhHMA4	32.9^{b}	4.7	13.9^{a}	0.8	71.9^{a}	2.8	199.2^{b}	4.3	52686.2^{ac}	1023.8	43640.1^{bd}	591.3	6178.5^{bc}	140.2
AhHMA4HA	40.3^{b}	1.1	14.3^{a}	0.8	78.1^{a}	2.3	196.1^{b}	2.4	49576.4^{bc}	976.3	47440.2^{c}	820.9	6247.9^{ac}	99.3
AhHMA4CCAA	20.0^{a}	1.3	15.0^{a}	0.6	69.0^{a}	1.9	222.7^{a}	3.8	54349.8^{a}	963.8	41985.4^{ad}	956.2	6833.9^{d}	130.0

B. Root

Genotype	Zn		Cu		Fe		Mn		Ca		K		Mg	
	Mean	SEM	Mean	SEM	Mean	SEM	Mean	SEM	Mean	SEM	Mean	SEM	Mean	SEM
hma2hma4	483.4^{a}	28.4	151.6^{a}	8.8	1230.5^{a}	81.1	316.5^{a}	124.4	5812.8^{a}	279.4	61049.4^{a}	509.7	1910.3^{a}	60.6
AhHMA4	292.4^{b}	18.0	132.8^{a}	18.5	952.2^{a}	88.0	254.9^{a}	74.5	5323.0^{ac}	85.0	63592.6^{a}	1374.6	1960.9^{a}	35.7
AhHMA4HA	307.1^{b}	11.7	124.3^{a}	15.7	1031.2^{a}	171.1	255.1^{a}	63.9	5247.2^{ac}	85.5	61774.5^{a}	2368.9	1878.2^{a}	47.9
AhHMA4CCAA	416.4^{a}	25.1	143.6^{a}	15.7	1047.8^{a}	99.6	369.3^{a}	62.3	4991.2^{bc}	100.7	60872.3^{a}	1963.5	1806.1^{a}	36.1

Non-transformed *hma2hma4* mutant and expressing the native or mutant *A. halleri* HMA4 proteins under the control of *AtHMA4* promoter were grown for the last three weeks before harvest in Hoagland hydroponic medium containing 0.2 µM zinc. Mineral concentrations were measured in shoot (A) and root (B) tissues collected from two plants per line. Values (ppm) are means ± SEM of 2 independent lines from 3 biological replicates. The data were analyzed with a one-way ANOVA test followed by Tukey's multiple comparison tests. Statistically significant differences ($P < 0.05$) between means for each element are indicated by different superscripted letters. Ah: *A. halleri*; HA: His- --> Ala-stretch; CCAA: di-Cys --> di-Ala motifs.

```
AtHMA4       LREKKKIGNKKCYRASTSKLNGRKLEGDDDYVVDLEAGLLTKSGNGQCKSSCCGDKKNQE 762
AhHMA4       LREKKKIGNKKCYRASTSMLNGRKLEGDDDDAVDLEAGLLTKSGNGQCKSSCCGDKKNQE 762
AtHMA2       LSDKHKTGNK-CYRESSSSSVLIAEKLEGDAAGDMEAGLLPKISDKHCKPGCCGTKTQEK 752

AtHMA4       NVVMMKPSSKTSSDHSHPGCCGDKKEEKVKPLVKDGCCSEKTRKSEGDMVSLSSCKKSSH 822
AhHMA4       KVVMMKPSSKTSSDHSHPGCCGDKKQGNVKPLVRDGGCSEETRKAVGDMVSLSSCKKSSH 822
AtHMA2       AM----KPAKASSDHSHSGCCETKQKDNVTV-VKKSCCAEPVD----------------- 790

AtHMA4       VKHDLKMKGGSGCCASKNEKGKE---VVAKSCCEKPKQQVESVGDCKSGHCEKKKQAEDI 879
AhHMA4       VKHDLKMKGGSGCCANKSEKVEG---VVAKSCCEKPKQQMESAGDCKSSHCEEKKHAEEI 879
AtHMA2       ----LGHGHDSGCCGDKSQQPHQHEVQVQQSCHNKPSG---------------------- 824

AtHMA4       VVPVQIIGHALTHVEIELQTKETCKTSCCDSKEKVKETGLLLSSENTPYLEKGVLIKDEG 939
AhHMA4       VLPVQMIGQALTGLEIELQTKETCKTRCCDNKEKAKKKGLLLSSEDTSYLEKGVLIKDEG 939
AtHMA2       -----------------------LDSGCCGGKSQQ------------------------- 836

AtHMA4       NCKSGSENMGTVKQSCHEKGCSDEKQTGEITLASEEETDDQDCSSGCCVNEGTVKQSFDE 999
AhHMA4       NCKSACQKTGTVKESCHEKAPL-------------------------------------- 961
AtHMA2       ------PHQHELQQSCHDKPS--------------------------------------- 851

AtHMA4       KKHSVLVEKEGLDMETGFCCDAKLVCCGNTEGEVKEQCRLEIKKEEHCKSGCCGEEIQTG 1059
AhHMA4       ------------------DIETKLVSCGNTEGEVGEQTDLEIKIEGDCKSGCCSDEKQTG 1003
AtHMA2       ----------GLD----------------------------------------------- 854

AtHMA4       EITLVSEEETESTNCSTGCCVDKEEVTQTCHE---------------------------- 1091
AhHMA4       EITLASEEETDSTDCSSGCCMDKEEVTQICGLETEGGGDCKSHCCGTGLTQEGSSKLGNV 1063
AtHMA2       --------------------------------------------IGTGPKHEGSSTLVNL 870

AtHMA4       --------------------KPASLVVSGLEVKKDEHCESSHRAVKVETCCKVKIPEACA 1131
AhHMA4       ESAQSGGCGTVKVSSQSCCTSSTDLVLSDLQVKKDEHCKSSHGAVKVETCCKVKIPEACA 1123
AtHMA2       EGD---AKEELKVLVNGFCSSPADLAITSLKVKSDSHCK--------------------- 906

AtHMA4       SKCRDRAKRHSGKSCCRSYAKELCSHRHHHHHHHHHHHHVSA     1172
AhHMA4       PECKEKEKRHSGKSCCRSYAKEFCSHRHHHHHHHH-HHVSA     1163
AtHMA2       SNCSSRERCHHGSNCCRSYAKESCSHDHHHTRAHGVGTLKEIVIE  951
```

Figure S1. Amino acid sequence alignment of plant P_{IB-2} ATPase C-terminal extensions. The di-Cys motifs are highlighted in cyan. The alignment was obtained using Clustal Omega with default settings (Sievers *et al.*. 2011). At: *A. thaliana*; Ah: *A. halleri*. (Sievers F, Wilm A, Dineen D, *et al.* 2011. Fast, scalable generation of high-quality protein multiple sequence alignments using Clustal Omega. *Molecular Systems Biology* **7**, 539.)

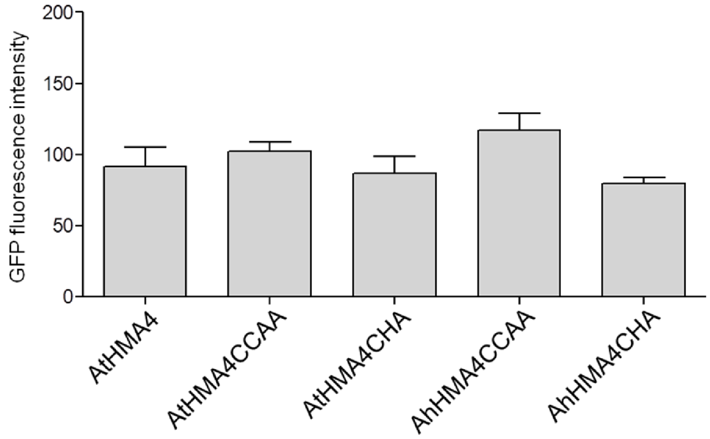

Figure S2. HMA4 native and variant protein expression level in *A. thaliana*. GFP fusions of HMA4 variants were imaged by confocal microscopy in roots of 18 day-old T1 seedlings. The native AtHMA4 and variant AxHMA4CCAA and AxHMA4CHA proteins are expressed in *A. thaliana* under the control of the *pAhHMA4-2* promoter. Protein expression levels were estimated through quantification of GFP fluorescence in imaged roots. For each genotype. values are mean ± SEM from three to six independent lines. The data were analyzed with a one-way ANOVA test followed by Tukey's multiple comparison tests. No statistically significant differences were detected. At: *A. thaliana*; Ah: *A. halleri*; CCAA: di-Cys --> di-Ala motifs; CHA: His- --> Ala-stretch and CCAA mutations combined.

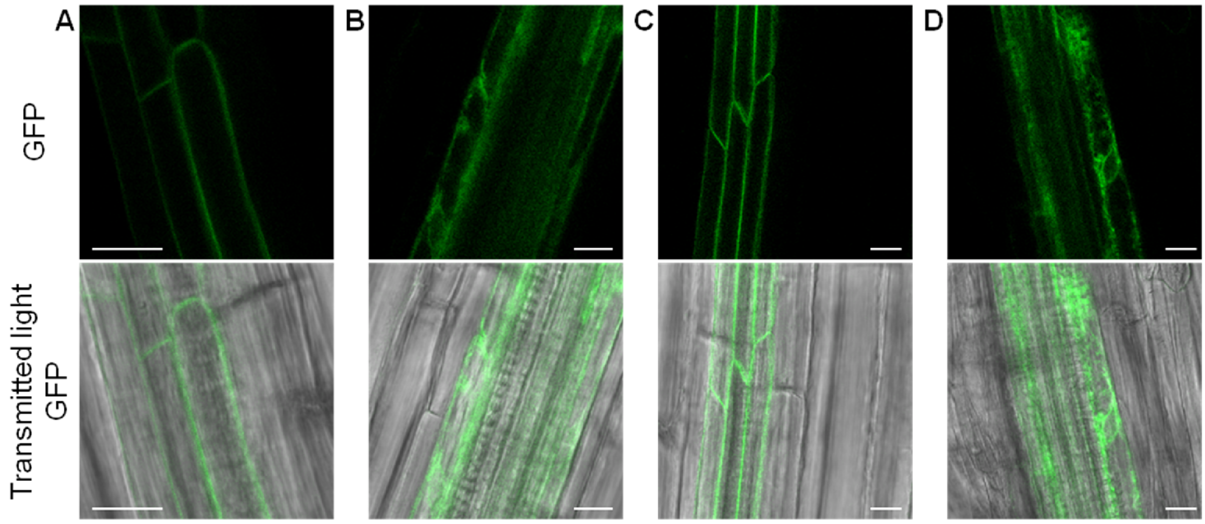

Figure S3. Closer views of HMA4 variant localization in *A. thaliana*. The AtHMA4CCAA (A). AtHMA4Ctrunc (B). AhHMA4CCAA (C) and AhHMA4Ctrunc (D) variants fused to GFP and expressed under the control of the copy 2 *AhHMA4* promoter in Col-0 were imaged by confocal microscopy in roots of 18 day-old T1 seedlings. The images are. for each genotype. representative of two to four independent lines from two independent experiments. Scale bars 10 µm. At: *A. thaliana*; Ah: *A. halleri*; CCAA: di-Cys --> di-Ala motifs; Ctrunc: fully truncated C-terminal extension.

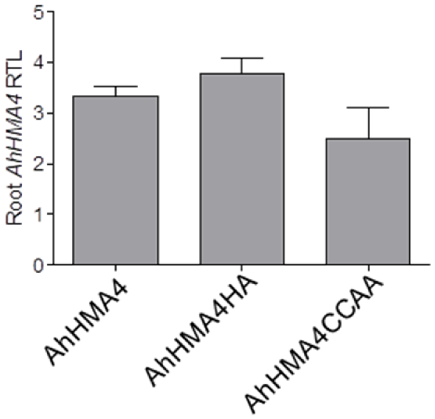

Figure S4. *AhHMA4* native and variant gene expression levels in complemented plants grown in hydroponic condition. *hma2hma4* mutant plants expressing the native AhHMA4 or mutant AhHMA4HA and AhHMA4CCAA proteins under the control of *pAtHMA4* were grown for the last three weeks before harvest in Hoagland hydroponic medium containing 0.2 µM zinc. Transcript levels were quantified from plant tissues collected from two plants per line. Relative transcript levels (RTL) of *AhHMA4* in roots are mean ± SEM of 2 independent lines from 2 biological replicates. The data were analyzed with a one-way ANOVA test followed by Tukey's multiple comparison tests. No statistically significant differences were detected. Ah: *A. halleri*; HA: His- --> Ala-stretch; CCAA: di-Cys --> di-Ala motifs.
